# Variation on a technique for the intra-muscular insertion of nerve endings to minimise neuropathic and residual pain in lower limb amputees: a retrospective cohort study

**DOI:** 10.1007/s00590-022-03281-4

**Published:** 2022-05-25

**Authors:** Victor Lu, Andrew Zhou, Matija Krkovic

**Affiliations:** 1 School of Clinical Medicine, University of Cambridge, Cambridge, CB2 0SP UK; 2 Department of Trauma and Orthopaedics, Addenbrooke’s Hospital, Cambridge, CB2 0QQ UK; 3 Christ’s College, St. Andrew’s Street, Cambridge, CB2 3BU UK

**Keywords:** Amputation, Phantom limb pain, Residual limb pain, PROMIS

## Abstract

**Introduction:**

A major cause of morbidity in lower limb amputees is phantom limb pain (PLP) and residual limb pain (RLP). This study aimed to determine whether a variation of the surgical technique of inserting nerve endings into adjacent muscle bellies at the time of lower limb amputation can decrease the incidence and severity of PLP and RLP.

**Methods:**

Data were retrospectively collected from January 2015 to January 2021, including eight patients that underwent nerve insertion (NI) and 36 that received standard treatment. Primary outcomes included the 11-point Numerical Rating Scale (NRS) for pain severity, and Patient-Reported Outcomes Measurement Information System (PROMIS) pain intensity, behaviour, and interference. Secondary outcome included Neuro-QoL Lower Extremity Function assessing mobility. Cumulative scores were transformed to standardised *t* scores.

**Results:**

Across all primary and secondary outcomes, NI patients had lower PLP and RLP. Mean ‘worst pain’ score was 3.5 out of 10 for PLP in the NI cohort, compared to 4.89 in the control cohort (*p* = 0.298), and 2.6 out of 10 for RLP in the NI cohort, compared to 4.44 in the control cohort (*p* = 0.035). Mean ‘best pain’ and ‘current pain’ scores were also superior in the NI cohort for PLP (*p* = 0.003, *p* = 0.022), and RLP (*p* = 0.018, *p* = 0.134).

Mean PROMIS *t* scores were lower for the NI cohort for RLP (40.1 vs 49.4 for pain intensity; *p* = 0.014, 44.4 vs 48.2 for pain interference; *p* = 0.085, 42.5 vs 49.9 for pain behaviour; *p* = 0.025). Mean PROMIS *t* scores were also lower for the NI cohort for PLP (42.5 vs 52.7 for pain intensity; *p* = 0.018); 45.0 vs 51.5 for pain interference; *p* = 0.015, 46.3 vs 51.1 for pain behaviour; *p* = 0.569). Mean Neuro-QoL *t* score was lower in NI cohort (45.4 vs 41.9; *p* = 0.03).

**Conclusion:**

Surgical insertion of nerve endings into adjacent muscle bellies during lower limb amputation is a simple yet effective way of minimising PLP and RLP, improving patients’ subsequent quality of life. Additional comparisons with targeted muscle reinnervation should be performed to determine the optimal treatment option.

## Introduction

Postamputation pain remains a common yet problematic complication following lower limb amputation. There are two main painful neurologic sequelae following an amputation: residual limb pain (RLP) and phantom limb pain (PLP), both of which are difficult to treat, lowers the patient’s quality of life and is a major factor for poor prosthetic function [1]. Prevalence rates are as high as 95% for experiencing one or more amputation-related pain [2], 76% for residual pain, and 85% for phantom pain [3].

PLP is an uncomfortable or painful sensation in the lost limb, either generalised to the whole limb, or one specific region of the lost limb [2]. Reported sensations are highly varied, including exteroceptive-type sensations (e.g. knifelike pain), nociceptive sensations (e.g. dull, cramping pain), and neuropathic sensations (e.g. electric-like pain). The sensations can also change over time, with one early study reporting a knifelike pain initially over the whole limb, changing to a burning pain localised to the distal aspect of the amputated limb after six months [4]. Initially thought to be a psychiatric illness, it is now understood to be due to cortical reorganisation, whereby cortical areas representing the amputated limb are overtaken by neighbouring areas in the somatosensory and motor cortices. Specifically, the greater the reorganisation in the somatosensory cortex, the more intense PLP becomes [5].

RLP, also known as ‘stump pain’, is localised to the remaining limb. Often described as an electric-like or burning pain, it can be perceived to be superficial at the amputation site, deep with the limb, or involving the whole limb. RLP is usually responsible for somatosensory discomfort in amputees immediately after amputation [6], whilst PLP is more a long-term consequence of amputation. Furthermore, there is correlation between the intensity of RLP and PLP, however this was only reported in a cohort of upper limb amputees [7].

RLP is mainly caused by terminal neuromas that from at the end of cut nerve endings. Many treatment options exist, from the early strategies utilising neuroablation via neurotoxins, to surgical management involving neuroma excision and translocating the nerve ending to another more favourable microenvironment. A new technique, targeted muscle reinnervation, was pioneered in 2002 by Dumanian, initially for better usage of myoelectric-controlled prosthesis [8]. This involves coapting the healthy nerve endings after neuroma excision to the distal aspect of a dissected motor nerve that has been made vestigial post-amputation. However, we noticed that patients treated with the simpler method of longitudinally dissecting the nerve in half, followed by directing the nerve endings into an adjacent muscle belly had better prosthesis usage and lowered amputation-related chronic pain, compared to patients who received standard treatment. We hypothesised that intramuscular insertion of dissected nerve endings performed at the time of amputation leads to reduced intensity of RLP and PLP and improved lower limb function compared to the standard treatment of lower limb amputees.

## Methods

This study was approved by the Cambridge University Hospitals institutional review board (project number 10157). The electronic patient records was searched between January 2015 and January 2021 for patients who had a lower limb amputation. Patients with upper limb amputations, younger than 18, hemipelvectomies, and those that were using neuromodulator medications were excluded. In total, 102 patients were individually contacted to complete questionnaires either via phone call or email. Twelve patients with less than 12 months follow-up were excluded. 56 patients were excluded due to incomplete responses or no response. Eight patients with amputation of digits rather than major limbs were excluded. The final sample size of general amputees was 36.

At the discretion of the surgical team, several patients with various indications for major lower limb amputation (trauma, vascular, neoplastic, infected and unsalvageable joint replacements) who had an amputation between January 2015 and January 2021 were given the option to concurrently undergo intramuscular insertion of nerve endings. Patients younger than 18, those with open wounds, those involved in other pain studies, those undergoing radiotherapy were excluded. Eight patients were identified, and all underwent nerve ending insertion in the same operative session as the amputation.

PLP and RLP pain severity were assessed using the numeric rating scale (NRS), which includes an eleven point Likert scale. Pain severity was assessed at its best, worst, and current level over a seven day period. NRS scores of ≤ 3 suggests mild pain, 4–6 suggests moderate pain, and ≥ 7 suggests severe pain. Ordinal logistic regression was performed to determine the odds of having worse pain in the nerve insertion (NI) group compared to the control group for both PLP and RLP.

Pain was further investigated using the Patient-Reported Outcome Measurement Information System (PROMIS) pain intensity short-form 3a, PROMIS pain behaviour short-form 7a, and PROMIS pain interference short-form 8a. The pain intensity questionnaire evaluates the patients’ pain over the past seven days using a five-point Likert scale ranging from ‘very severe pain’ to ‘no pain’. Pain behaviour looked at any manifestations of pain that would become noticeable to others, and used a six-point Likert scale from ‘never’ to ‘always’, whilst also including the option of ‘had no pain’. Grimacing, low mood, shouting for help are examples of outward expression of pain. Pain interference evaluated the extent to which pain affected patients’ daily life activities, and used a five-point Likert scale ranging from not at all to very much. Areas assessed included spending time with family and friends, doing household chores, and enjoying hobbies. Standardised t scores were calculated from cumulative scores using Assessment CenterSM’s HealthMeasures Scoring Service [9], with a higher *t* score suggest a greater pain intensity, greater pain interference or increased outward expression of pain. The aforementioned four questionnaires were given to both the normative sample of lower limb amputees, and amputees treated with insertion of nerve endings, assessing both RLP and PLP.

Functional assessment was conducted using Neuro-Quality of Life (Neuro-QoL) Lower Extremity Function (Mobility) Short Form, which contains eight items on a five-point Likert scale ranging from ‘without any difficulty’ to ‘unable to do’. Simple activities such as getting in and out of a car, pushing open a heavy door, walking for 15 min were assessed. Likewise, cumulative scores were converted to standardised *t* scores, with a higher score indicating better function.

### Surgical protocol

#### Nerve insertion group

When a neurovascular bundle is identified, the vessels are ligated and divided just short to the tip of the bone stump. Nerves were left approximately 5–10 cm longer than the maximal bone stump length, as observed grossly. The main nerves (i.e. tibialis posterior, sciatic nerve) were then divided longitudinally in half by sharp dissection leaving the fascicles intact as much as possible. This then gave two main branches of the nerve. For smaller nerves (deep and superficial peroneal nerve, saphenous and sural nerves), the nerves were left approximately 5–8 cm longer, but were not divided/split.

**Fig. 1:**
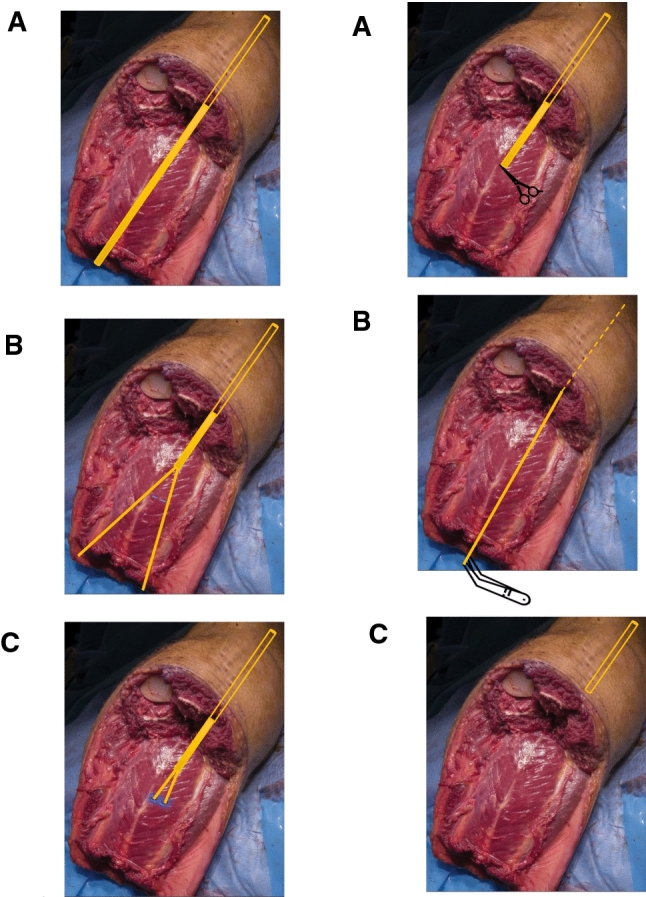
Surgical Protocol. Left (Nerve Insertion Group) **A** Nerves were left 5–10 cm longer than the maximum bone stump, and an adjacent muscle belly suitable for insertion was located. **B** Nerves were divided longitudinally in half by sharp dissection. Small slits are created in the muscle belly. **C** The length of the nerves were adjusted, and nerve endings were secured with sutures. The needle of the suture was inserted into the muscle belly and exited on the opposite side, securely burying the nerve end inside the muscle. Right (Control Group) **A** A neurovascular bundle is identified, and nerve endings are dissected proximally. **B** Nerve endings are placed under traction and secured with a clamp at the most distal point. **C** Nerve is allowed to freely retract into the residual limb

After the nerves were divided and muscle bellies prepared for suturing to the bone stump, the nerve lengths were carefully trimmed so that they fit in the adjacent muscle belly and will not be kinked when the stump is formed. Just before the muscle bellies were sutured to form the stump, the nerve endings were secured with 2/0 absorbable suture, and a small slit was created in the muscle belly. The needle of the suture was then inserted into the muscle belly and exited on the opposite side, securely burying the nerve end inside the muscle. When the suture exits the muscle belly on the other side, the suture was secured to the muscle. For each separate nerve ending, the procedure was repeated. The purpose of this procedure is to allow nerve growth into the adjacent muscle belly to avoid (or limit) neuroma growth. A diagram is shown in Fig. 1.

#### Control group

After identifying a neurovascular bundle, the nerve endings were dissected proximally such that eventually, the resulting end would lie deep in the stump, away from the distal end of the residual limb. The nerves were then placed under some traction, but care was taken to avoid excessive traction which could cause proximal pain and neuropathy. Ligatures were placed on the nerve ends, carefully avoiding nearby vessels, as ligation with pulsing vessels can become a source of throbbing and pain. The nerve was then allowed to freely retract.

### Statistical analysis

Statistical analysis was performed using IBM SPSS Statistics version 27 (IBM Corp, Armonk, NY, USA). Fischer’s exact test (*n* < 10) or Chi-squared test (*n* ≥ 10) were used for categorical values, and Mann–Whitney U test for nonparametric continuous variables. Bonferroni’s adjustment was applied to reduce the familywise error rate when performing multiple comparisons of primary outcomes. Statistical significance was set to *p* ≤ 0.05.

## Results

Table 1 presents demographical details of the patients included in this study. All patients had a follow-up longer than 12 months; the average follow-up for the NI cohort is 74 weeks, and the average follow-up for the control cohort is 107 weeks. Trauma was the most common cause of amputation in both cohorts (87.5% for NI cohort and 77.8% for control cohort). Below knee amputations accounted for the majority (62.5% for NI cohort and 55.6% for control cohort). All eight patients in the NI cohort underwent the procedure on the same day as the amputation.

**Table 1:** Patient demographics

Characteristic	Nerve Insertion (*n* = 8)	General (*n* = 36)
Age	45.4 (SD 20.9)	48.7 (SD 14.4)
Male Gender (%)	5 (62.5)	23 (63.9)
*Race*		
White	7	27
Asian	0	1
African American	0	3
Hispanic	1	4
Other	0	1
*Occupational Status*		
Employed with wages	4	22
Self-employed	2	6
Student	1	0
Unable to work	1	0
Retired	0	8
*Location of amputation*		
Above knee	2	14
Below knee	5	20
Through knee	1	2
*Time since amputation*		
1–2 years	5	3
2–3 years	3	10
3–4 years	0	18
4–5 years	0	5
*Cause of amputation*		
Trauma	7	28
Infection	1	6
Ischaemia	0	2
Tumour	0	0

Patient-reported NRS scores are presented in Table 2. Those with *p* values ≤ 0.05 are shown in bold. Mean ‘worst pain’ score was 3.5 out of 10 for PLP in the NI cohort, compared to 4.89 in the control cohort (*p* = 0.298), and 2.6 out of 10 for RLP in the NI cohort, compared to 4.44 in the control cohort (*p* = 0.035). Mean ‘best pain’ and ‘current pain’ scores were also superior in the NI cohort for PLP (*p* = 0.003, *p* = 0.022), and RLP (*p* = 0.018, *p* = 0.134), although the improvement in current pain NRS score did not reach statistical significance. At the very worst, 33.3% in the control cohort had severe PLP and 16.7% had severe RLP, compared with 0% and 12.5% in the NI cohort, respectively. Notably, no patients in the NI cohort reported any PLP or RLP at their best state. No patients in either the NI or control group suffer from severe PLP or RLP in their current state, and only mild PLP or RLP was reported from patients in the NI cohort in their current state. Ordinal logistic regression analysis determined that compared to NI patients, the control group patients had a 2.097 higher odds (95% CI: 0.534 to 8.245) and 5.742 higher odds (95% CI: 1.362 to 24.201) of increased PLP and RLP, respectively.

**Table 2:** NRS Pain level scores

	Nerve Insertion (n = 8)		General (n = 36)		*p* value
	Mean (± SD)	Range	Mean (± SD)	Range	
*PLP NRS (0–10)*					
Best	0 (± 0)	0–0	1.42 (± 1.6)	0–6	**0.003**
Worst	3.5 (± 2.4)	0–7	4.89 (± 2.4)	1–9	0.320
Current	0.75 (± 1.2)	0–3	2.11 (± 1.6)	0–6	**0.022**
*RLP NRS (0–10)*					
Best	0 (± 0)	0–0	0.81 (± 1.0)	0–3	**0.018**
Worst	2.6 (± 2.4)	0–7	4.44 (± 2.1)	0–8	**0.035**
Current	0.75 (± 1.2)	0–3	1.56 (± 1.5)	0–6	0.134

As shown in Table 3, compared to the control cohort, mean PROMIS *t* scores were lower for the NI cohort for RLP (40.1 vs 49.4 for pain intensity; *p* = 0.014, 44.4 vs 48.2 for pain interference; *p* = 0.085, 42.5 vs 49.9 for pain behaviour; *p* = 0.025). Mean PROMIS *t* scores were also lower for the NI cohort for PLP (42.5 vs 52.7 for pain intensity; *p* = 0.018); 45.0 vs 51.5 for pain interference; *p* = 0.015, 46.3 vs 51.1 for pain behaviour; *p* = 0.569). Table 4 shows the mean Neuro-QoL *t* score was higher in the NI cohort than control cohort (45.4 vs 41.9; *p* = 0.032). Those with *p* values ≤ 0.05 are shown in bold.

**Table 3:** PROMIS pain scores for intensity, behaviour, and interference

	Nerve Insertion (n = 8)		General (n = 36)		*p* value
	Mean (± SD)	Range	Mean (± SD)	Range	
*PLP PROMIS t scores*					
Intensity	42.5 (± 9.3)	36.3–58.9	52.7 (± 10.0)	36.3–66.8	**0.018**
Interference	45.0 (± 6.0)	40.7–54.9	51.5 (± 6.6)	40.7–62.8	**0.015**
Behaviour	46.3 (± 13.2)	36.7–64.2	51.1 (± 7.5)	36.7–57.9	0.569
*RLP PROMIS t scores*					
Intensity	40.1 (± 5.4)	36.3–49.0	49.4 (± 10.0)	36.3–67.8	**0.014**
Interference	44.4 (± 5.1)	40.7–51.7	48.2 (± 5.9)	40.7–56.4	0.085
Behaviour	42.5 (± 8.0)	36.7–52.6	49.9 (± 9.3)	36.7–61.4	**0.025**

**Table 4:** Neuro-QoL Scores for Lower Limb Mobility

	Nerve Insertion (n = 8)		General (n = 36)		p value
	Mean (± SD)	Range	Mean (± SD)	Range	
Neuro-QoL Lower Limb Mobility *t* Score	45.4 (± 3.6)	40.5–50.6	41.9 (± 5.2)	32.5–58.6	**0.032**

## Discussion

There is mounting evidence that targeted muscle reinnervation (TMR) leads to reduced PLP and RLP, and improved lower limb function and mobility, when compared to amputees treated conventionally [8, 10–12]. TMR involves identifying small motor nerves innervating adjacent muscle fascicles, and coapting them to major mixed or sensory nerves that have been severed by the amputation. There are specific donor-target nerve combinations for various kinds of amputations published in the literature [13–15]. Criticisms of TMR include the fact that it causes unnecessary surgical insult and adds at least an extra hour of surgical time [12]. There is widespread evidence that increased operative time leads to increased post-operative infection rate, with a 60 min increase leading to a 37% increased likelihood of surgical site infections [16–20]. Increased operative time can also lead to increased hospital length of stay [21]. TMR also requires a dedicated team knowledgeable in the field of nerve transfers, and can only realistically be done in specialist major trauma centres. TMR can also be inappropriate when dealing with nerves of different sizes, whereby coaptation of these nerves leads to axonal escape, which is an independent risk factor for neuronal formation [22]. Furthermore, some studies have reported that TMR has failed to improve myoelectric prosthetic control, with a high rate of abandonment [23]. The purpose of our study was to seek improvements in pain control and decrease symptomatic neuroma formation, rather than seeking to create separate electromyography signals feasible for prosthesis control.

Another technique that has gained traction is regenerative peripheral nerve interface (RPNI), which involves the insertion of a proximally dissected end of a peripheral nerve into a free muscle graft [22]. The theory behind the elimination of painful neuromas is similar to our study, whereby the muscle belly provides a physiological endpoint that limits neuroma formation. Nevertheless, RPNI involves a certain degree of surgical complexity, requires expendable muscles for denervation and grafting, of which there is a paucity in some areas such as the hand, and issues related to graft failure may cause unwanted sequelae [22]. This study therefore investigates a simpler and more straightforward alternative for reducing PLP and RLP in amputees. When performed concurrently at the time of lower limb amputation, nerve ending insertion into adjacent muscle bellies markedly decreases PLP and RLP intensity. The NI cohort saw better daily experiences of pain, by virtue of improvements in pain interference and pain behaviour. Finally, the NI cohort experienced markedly better lower limb mobility which helped to improve their quality of life.

Compared to the control group, NI decreased PLP by an average of 1.39 points (28.4%) on the NRS scale and decreased RLP by 1.84 points (41.4%). Studies of both acute and chronic pain suggest that a reduction of 2 points on the NRS scale or a decrease in approximately 30% represents a clinically important difference and is associated with decreased need to take pain relieving medication [24]. Significantly, all patients who underwent NI experienced a period of time without any PLP or RLP, whilst in the control cohort, only 36% experienced a period of no PLP, and 52.8% had an episode free of RLP. Regarding current levels of pain, the NI cohort achieved a PLP-free rate 2.5 times that of the control cohort (62.5% vs 25.0%), and a RLP-free rate 1.73 times that of the control cohort (62.5% vs 36.1%). Nevertheless, these figures are far superior than the pain-free levels reported in the literature which are as low as 9% [25].

PROMIS pain intensity, behaviour, and interference scores further supports the efficacy of NI into adjacent muscle belly. Nevertheless, despite improvements in pain behaviour in the NI cohort, the difference with the control cohort was not statistically significant. As reported in a clinical validity study, PROMIS pain behaviour seems to be less sensitive to pain intervention than pain interference scores [26]. This probably reflects the fact that pain behaviour is multifactorial, and learned responses to pain takes a significant period of time to change. The improvement in PROMIS pain interference in the NI cohort also failed to reach statistical significance, perhaps due to the low average scores in both cohorts and the relatively small sample size. Given the significant improvements in pain scores, it was not surprising that the NI cohort experienced better lower limb mobility and function compared to the control cohort. Pain provides a unique domain to functional impairment, that is independent of psychological factors or physical impairments [27].

The surgical protocol of our nerve insertion cohort is a variation of previously described protocols that implanted proximal nerve endings into adjacent muscles [28, 29]. Our protocol involved the additional step of longitudinally dividing major nerves in half prior to insertion. There is a paucity of literature describing the benefits and physiological underpinnings of this procedure, and no study has been performed that compares this to other surgical protocols such as TMR or RPNI; however, the results of our study suggest that this is a technique worth further investigating. One comparative study performed histological assessment and concluded that regarding neuroma and scar tissue formation, muscle implantation is superior to simply proximally cutting the nerve and allowing it to retract [30]. The theoretical rationale is that a painful neuroma is created from the directionless proliferation of a transected axon and its associated Schwann cells, fibroblasts, and vasculature. With nerve insertion into a neighbouring muscle belly, rather than forming a neuroma, the inserted nerve endings would arborise along underlying intramuscular motor pathways in an organised fashion, leading to the creation of functional neuromuscular channels [31]. Longitudinally dissecting major nerves prior to insertion helps to facilitate this process by creating a milieu for more efficient arborisation, and reducing the impact of physical and chemical axon regeneration inhibitors, which decreases the chance of haphazard axonal proliferation [31, 32]. Yet it is still unclear how the interaction of regeneration axons with the myogenic environment leads to decreased neuroma pain. Unlike the study by Pet et al. [29], our protocol did not involve denervation of muscle bellies prior to nerve insertion, in order to reduce the chance of muscle atrophy. Nevertheless, Bain et al. suggested that sensory nerve reinnervation can protect a denervated lower limb muscle from atrophy and maintain functional capacity [33], yet this was only examined in rats.

Our study has several limitations. As with most studies of this format, the main limitation is the reliance of our results on patients’ active recall, judgement of one’s own condition, and understanding of the questionnaires. The 11-point NRS questionnaire, PROMIS measures, and Neuro-QoL assessment were chosen based on their widespread use and proven clinical validity across multiple specialties [26, 34–37]. All these questionnaires represent a snapshot in time, and relies on a robust 7-day recall period. The use of drugs such as opioids could act as a proxy of pain medication usage and reflect the wellbeing of the patient, however we elected to not include this data since we found that drug compliance was overall quite poor, and patients could very likely have obtained extra painkillers over the counter. Despite being demographically similar to the control group, the small sample size of the NI cohort may preclude the ability to detect certain differences in pain scores, and reduces the impact of any p values < 0.05. Furthermore, imaging was not performed to confirm the absence/presence of neuroma. Neuromas could have formed asymptomatically, and the superior pain outcomes in the NI cohort could indicate proximal growth of neuromas away from the impact of the stump. We were unable to simultaneously recruit patients who were treated with TMR, which would have created an ideal three-way comparison between amputees receiving normal treatment, TMR patients, and patients undergoing NI into an adjacent muscle belly. Finally, we were not able to assess patients’ prosthetic usage, however the focus of this study was to focus on symptomatic neuroma prevention and pain control. We believe that our study acts as a springboard for further research into the optimum treatment for amputees suffering from acute and chronic pain.

## Conclusion

When performed in the same sitting as the amputation, nerve insertion into adjacent muscle bellies decreased PLP and RLP intensity, decreased pain interference and pain behaviour, and improved lower limb function and mobility. Given the increasing prevalence of PLP and RLP and the significant morbidity it causes, the simple and efficient technique of NI has the potential to have a major impact of many future amputees. TMR is undeniably an advancement for better myoelectric prosthesis control, however, NI is a simple and efficient technique for decreasing symptomatic neuroma formation in patients who do not urgently need TMR for myoelectric prosthesis.

In light of our results, we recommend that future studies using larger multi-centre cohorts investigate whether the benefits of NI over conventional treatment is similar to that of TMR, and if so, whether the shorter operative time, decreased surgical insult, and reduced surgical complexity warrants NI to be the mainstay of multidisciplinary amputee care.
